# p38 MAPK Inhibition Improves Synaptic Plasticity and Memory in Angiotensin II-dependent Hypertensive Mice

**DOI:** 10.1038/srep27600

**Published:** 2016-06-10

**Authors:** Hai-long Dai, Wei-yuan Hu, Li-hong Jiang, Le Li, Xue-feng Gaung, Zhi-cheng Xiao

**Affiliations:** 1 Department of Cardiology, Yan’an Affiliated Hospital of Kunming Medical University, Kunming, China; 2 Institute of Molecular and Clinical Medicine, Kunming Medical University, Kunming, China; 3 Department of Anatomy and Developmental Biology, Monash University, Clayton, Australia; 4 Department of Cardiothoracic Surgery, Yan’an Affiliated Hospital of Kunming Medical University, Kunming, China; 5 Department of outpatient, Ganmei Affiliated Hospital of Kunming Medical University, Kunming, China

## Abstract

The pathogenesis of hypertension-related cognitive impairment has not been sufficiently clarified, new molecular targets are needed. p38 MAPK pathway plays an important role in hypertensive target organ damage. Activated p38 MAPK was seen in AD brain tissue. In this study, we found that long-term potentiation (LTP) of hippocampal CA1 was decreased, the density of the dendritic spines on the CA1 pyramidal cells was reduced, the p-p38 protein expression in hippocampus was elevated, and cognitive function was impaired in angiotensin II-dependent hypertensive C57BL/6 mice. *In vivo*, using a p38 heterozygous knockdown mice (p38^KI/+^) model, we showed that knockdown of p38 MAPK in hippocampus leads to the improvement of cognitive function and hippocampal synaptic plasticity in angiotensin II-dependent p38^KI/+^ hypertensive mice. *In vitro*, LTP was improved in hippocampal slices from C57BL/6 hypertensive mice by treatment with p38MAPK inhibitor SKF86002. Our data demonstrated that p38 MAPK may be a potential therapeutic target for hypertension-related cognitive dysfunction.

Cognitive decline is rapidly becoming an important cause of disability worldwide and contributes significantly to increased mortality. Cardiovascular risk factors, particularly hypertension, play an important role in the development of cognitive dysfunction, including Alzheimer’s disease (AD), leading to increased prevalence of dementia in the aging population^1^,^2^,^3^. Heart–brain interaction is one of the 10 most-promising advances in cardiovascular therapies and interventions for the next decade^4^. However, the relationship between hypertension and cognitive function is complex and not completely understood, the results of clinical trials of anti-hypertension therapies on cognitive function are not all consistent and limited by the short-term follow up^1^,^5^,^6^.

The hippocampus is thought to be an important region of the brain for learning and memory. It plays important roles in the consolidation of information from short-term memory to long-term memory and spatial navigation. Synaptic plasticity is the neurobiological basis of learning and memory, which includes functional plasticity of synaptic transmission and structural plasticity of dendritic spine morphology and number^7^,^8^. Long-term potentiation (LTP) is an important manifestation of functional plasticity of synaptic transmission, mostly intensely studies in the hippocampus, and is a widely studied as a cellular model of learning and memory^9^.

In recent decades, many reports have shown that p38 MAPK signalling pathway plays important roles in hypertension, cognitive dysfunction, and synaptic plasticity^10^,^11^,^12^,^13^. p38 MAPK inhibition can ameliorated angiotensin II–induced target organ damage and aortic stiffening^14^,^15^. Activated p38 MAPK was seen in human AD brain tissue and in AD-relevant animal models^11^,^16^,^17^,^18^. The block of LTP induced by synthetic Abeta was prevented by the p38 MAPK inhibitor^19^.

In this study, we investigated whether p38 MAPK mediate the deleterious effects of hypertension on cognitive function. Using angiotensin II-dependent hypertensive mice, we found that activation of p38 MAPK, synaptic plasticity impairment and memory disorder. *In vivo*, using a p38 heterozygous knockdown mice (p38^KI/+^) model, we found that synaptic plasticity and memory were improved in angiotensin II-dependent p38^KI/+^ hypertensive mice. *In vitro*, application of p38MAPK inhibitor SKF86002, we found that impaired LTP was improved in hippocampal slices from angiotensin II-dependent hypertensive mice. These data provide novel insights into the mechanisms of hypertension-related cognitive dysfunction and suggest new treatment strategies for counteracting the cognitive impairment of hypertension.

## Results

### Angiotensin II induces hypertension in both C57BL/6 and p38^kI/+^ Mice

**Figure 1:**
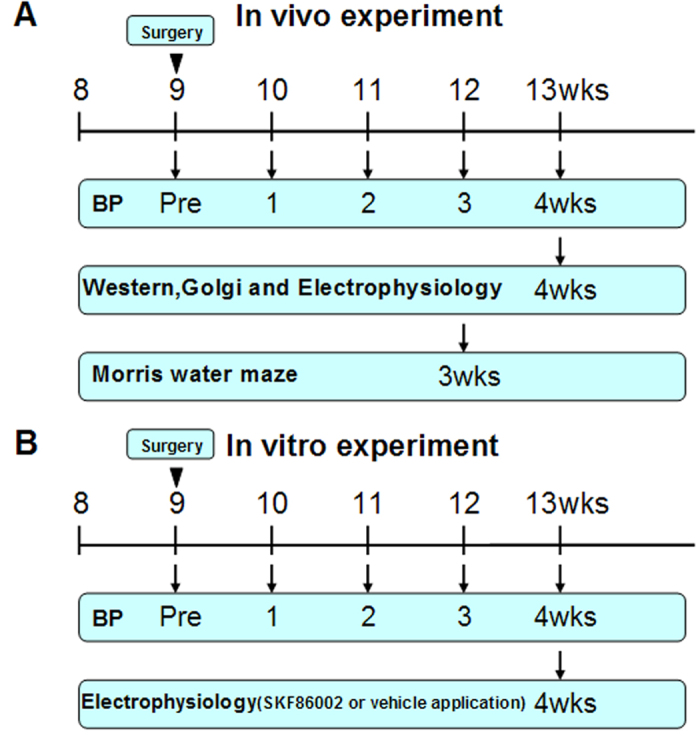
Schematic depiction of experimental design. (**A**) experimental design *in vivo*. (**B**) experimental design *in vitro*.

**Figure 2:**
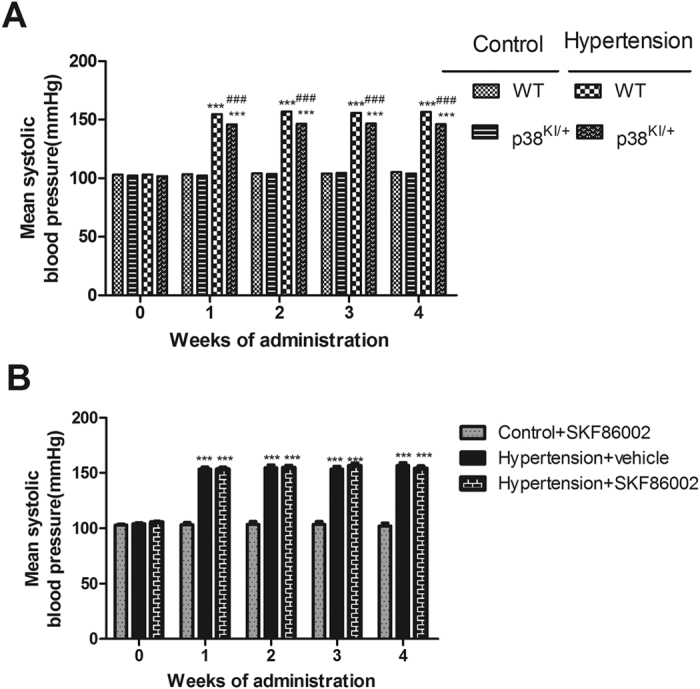
Mean systolic blood pressure during chronic angiotensin II or saline (Ctrl) perfusions. (**A**) *in vivo* experiment (n = 17–20, ***p < 0.001 vs control WT and p38^KI/+^ group, ^###^p < 0.001 vs HT group). (**B**) *in vitro* experiment, (n = 5, ***p < 0.001 vs control + vehicle group).

Angiotensin II treatment was started at the age of 9 weeks and was continued for 4 weeks. Schematic depiction of experimental design in Fig. 1. *In vivo* experiment, at 9 weeks, there was no significant difference of systolic blood pressure between control C57BL/6 and p38^KI/+^ mice. Angiotensin II treatment resulted in a rapid (1 weeks after implantation), significant, and sustained raise in systolic blood pressure. Although blood pressure in p38^KI/+^ &HT mice slightly lower than HT mice (p < 0.001), it remains at its higher level in both HT and p38^KI/+^ &HT mice compared to control (Fig. 2A). *In vitro* experiment, angiotensin II significantly raised the systolic blood pressure (p < 0.001), and there is no difference between hypertension + vehicle group and hypertension + SKF86002 group (Fig. 2B).

### p38 MAPK is inhibited in p38^KI/+^ &HT mice hippocampus

**Figure 3:**
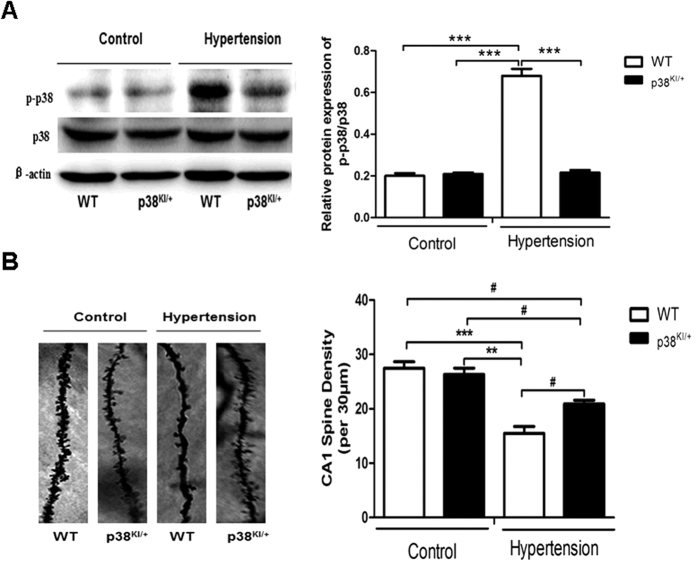
p38 MAPK knockdown increases dendritic spine number on hippocampal CA1 pyramidal cells. (**A**) WT and p38^KI/+^ mice were subjected to hypertension surgery at 9 weeks of age. Four wks post-surgery, western blotting was performed on the hippocampal lysates.Right bar graph shows quantification of p38 MAPK phosphorylation in hippocampus, n = 3 mice per experimental group. (**B**) Representative Golgi stain image. Right bar graph shows quantification of dendritic spine density, n = 3, and five neurons per mouse were analyzed. Data are presented as mean ± SEM. ***p < 0.001, **p < 0.01, ^#^p < 0.05 by one-way ANOVA with Bonferroni post-test.

To test our hypothesis that p38 MAPK critically regulates hypertension-related cognitive dysfunction, we examined the level of p38 MAPK activity (phosphorylation) in the hippocampus. Nine weeks old WT and p38^KI/+^ mice were subjected to hypertension surgery and hippocampal lysates were analyzed for p38 MAPK phosphorylation at four weeks post-surgery. As shown in Fig. 3A, the phosphorylation of p38 MAPK was significantly increased in the HT mice hippocampus compared to control hippocampus (p < 0.001), which was decreased in p38^KI/+^ &HT mice comparison to HT mice (p < 0.001), and there was no significant difference between WT mice and p38^KI/+^ mice.

### Dendritic spine number is increased in p38^KI/+^ &HT mice

To determine the effect of p38 MAPK knockdown on dendritic spine number, WT and p38^KI/+^ mice were subjected to hypertension at 9 weeks of age. Dendritic spine number on hippocampal CA1 pyramidal cells, was determined on week 4 post-surgery by Golgi analysis. As shown in Fig. 3B, the density of dendritic spines (number/30 μm) was decreased in HT mice comparison to control mice (p < 0.01), but which was increased in p38^KI/+^ &HT mice compared to HT mice (p < 0.05).

### p38 MAPK inhibition rescues hippocampal LTP deficit

**Figure 4:**
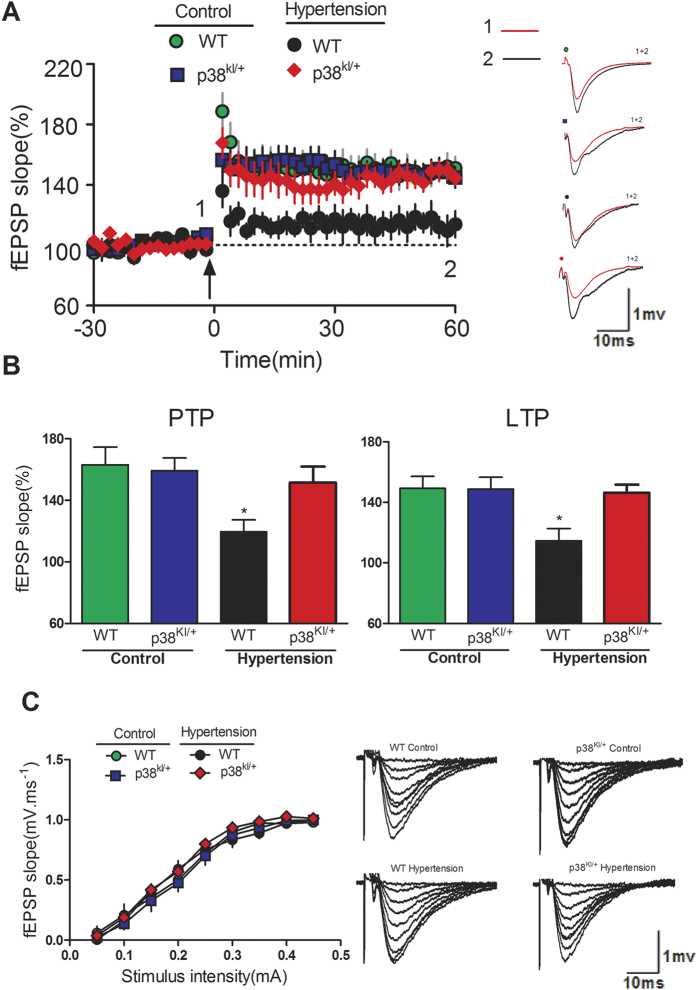
p38 MAPK knockdown rescues hippocampal LTP impairment. (**A**) LTP induced by TBS in four group mice. Insert is representative waves before and after TBS application. Right graph shows the traces in a represented fEPSPs recorded in a hippocampal slice from four group mice. (**B**) PTP and LTP data from four group were summarized and compared together, n = 8 slices from five animals for each group, *p < 0.05, HT group vs WT group or p38^KI/+^ group or p38^KI/+^ &HT group. (**C**) I/O curves are shown (all P > 0.05).

**Figure 5:**
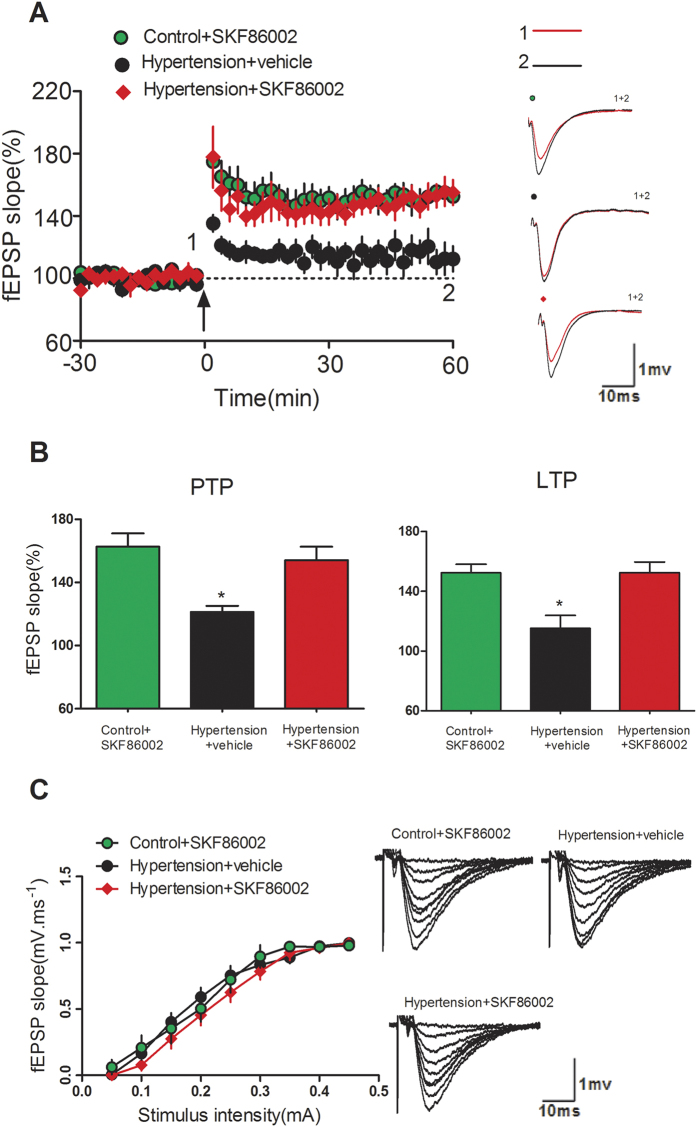
SKF86002 rescued hippocampal LTP deficit in hypertensive mice. (**A**) LTP induced by TBS in three group mice. Insert is representative waves before and after TBS application. Right graph shows the traces in a represented fEPSPs recorded in a hippocampal slice from three group. (**B**) PTP and LTP data from three group were summarized and compared together, n = 8 slices from five animals for each group, *p < 0.05, hypertension + vehicle group vs control + SKF86002 group or hypertension + SKF86002 group. (**C**) I/O curves are shown (all P > 0.05).

To investigate synaptic functional changes, we performed extracellular electrophysiological recordings on hippocampal slices at four weeks post-surgery. Hippocampal slices were incubated with artificial cerebrospinal fluid (ACSF) for 1 h and then LTP was detected after theta burst stimuli (TBS) delivery. Data of fEPSP slopes during 0–10 min (i.e. post-tetanic potentiation, PTP) and 50–60 min (i.e. LTP) after TBS application were summarized and compared. As shown in Fig. 4A,B, LTP induction was significantly impaired in slices of HT mice when compared with that in controls, while in slices of p38^KI/+^ &HT mice significantly rescued LTP induction compared to HT mice (p < 0.05). Furthermore, no unequivocal change was found for the I/O curves of the various treatment conditions. (all P > 0.05; Fig. 4C), indicating that these treatments did not alter basal synaptic transmission. *In vitro* experiment, p38MAPK inhibitor SKF86002 incubation significantly improved LTP induction in slices from hypertensive mice (p < 0.05)(Fig. 5A,B), and no unequivocal change was found for the I/O curves after the treatments of SKF86002 when compared with that after the vehicle treatment (all P > 0.05; Fig. 5C).

### p38 MAPK knockdown protects against memory deficit in p38^KI/+^ &HT mice

**Figure 6:**
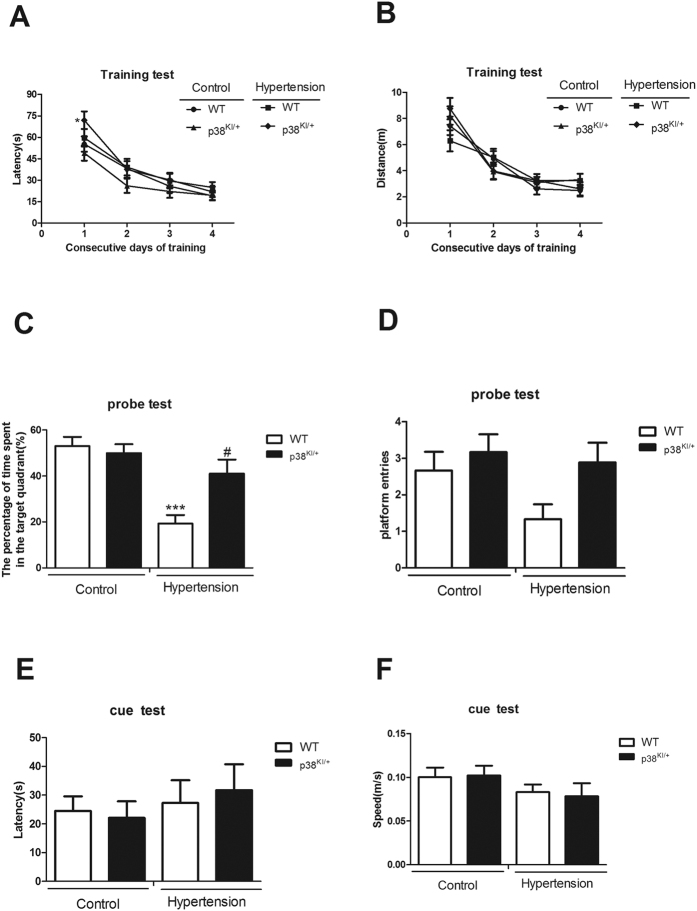
p38 MAPK knockdown prevents memory deficit, n = 9–12. (**A**) Latency taken to escape from the water in the platform trials. (**B**) Distance taken to escape from the water in the platform trials. (**C**) Percentage of time spent in the quadrant area relative to the total time spent in the pool in the probe trial. (**D**) The number of crosses over the exact location of the hidden platform in the probe trial. (**E**) Latency taken to escape from the water when the platform is visible in the cue test. (**F**) Swimming speed in the cue test. *p < 0.05, p38^KI/+^ &HT group vs p38^KI/+^ group; ***p < 0.001, HT group vs WT group or p38^KI/+^ group; ^#^p < 0.05, p38^KI/+^ &HT group vs HT group.

To investigate the influence of p38 MAPK knockdown on cognitive function, four group mice were subjected to the Morris water maze test at three weeks post-surgery. During the training days, the latency and distance for finding the escape platform were measured to assess spatial learning abilities. There were no differences in learning abilities among different groups, even though the latency in p38^KI/+^ &HT mice was longer than p38^KI/+^ mice in first training day (p < 0.05). (Fig. 6A,B). Because, the distance and other 3 days latency were no differences.

Memory was assessed by the probe test. The time spent in the target quadrant of HT mice was less compared to the control mice (p < 0.001), while p38^KI/+^ &HT mice spent more time in the target quadrant area than HT mice (p < 0.05). (Fig. 6C). p38^KI/+^ &HT mice entered the platform area more frequently than HT mice, but the difference between the two groups did not reach the statistical significance (Fig. 6D).

The cue test, where the latency is used to measure sight and speed to evaluate motricity, showed no significant difference among different groups (Fig. 6E,F).

## Discussion

In this study, we demonstrated that hippocampal p38 MAPK activation, synaptic plasticity impairment and memory disorder in angiotensin II-dependent hypertensive mice. Furthermore, we showed that impaired synaptic plasticity and memory were rescued in transgenic p38 MAPK knockdown hypertensive mice, and p38 MAPK inhibitor SKF86002 rescued hippocampal LTP deficit in slices from hypertensive C57BL/6 mice. This study provided evidence for p38 MAPK may be a potential therapeutic target for the treatment of hypertension-related cognitive dysfunction.

Hypertension is a risk factor for senile dementia. Many observational studies have shown that hypertension is a risk factor for cognitive impairment or dementia in the elderly^20^,^21^,^22^. In the largest and longest prospective study, involving 3,381 adults (aged 18–30 years at baseline) in four cities in the USA and with 25 years of follow-up, cognitive function was assessed at year 25 with the most-sophisticated technology, which showed that cumulative levels of blood pressure were associated with impaired cognition^2^. In hypertensive animal models, behavioural studies revealed cognitive impairment^23^,^24^,^25^,^26^,^27^. In our study, we found that learning ability was not impaired and that memory storage was affected in HT mice, this may be explained by the period of hypertension is not too long, and memory impairment is the earliest and most prominent clinical features^28^.

Although the relationship between hypertension and dementia is well established, the impact of antihypertensive treatment and management of other risk factors on cognition is less clear. Several studies have investigated the effect of antihypertensive treatment on the development of dementia in the hypertensive patients^29^,^30^,^31^,^32^,^33^. However, the results were markedly different among the studies, and meta-analyses have not reached a conclusion^34^,^35^. Furthermore, high SBP was associated with better cognitive performance in centenarians^36^,^37^. Deposition of β-amyloid in brain is enhanced in mouse models of arterial hypertension^38^,^39^. Howere, bapineuzumab and solanezumab, 2 Aβ targeting monoclonal antibodies, have failed to meet trial cognitive end points in mild to moderate AD patients^40^,^41^.

The hippocampus is essential for consolidation of declarative information and spatial navigation. Synaptic plasticity is an important cellular mechanism that regulates memory formation. LTP in the CA1 region of the hippocampus has been the primary model to study the cellular and molecular basis of synaptic plasticity. Alterations of dendritic spine morphology, changes of spine type ratios or density have consequently been found in paradigms of learning and memory, and accompany many neurodegenerative disease. A significant amount of evidence suggests that the p38 MAPK signalling cascade plays a crucial role in synaptic plasticity and in neurodegenerative diseases. p38 MAPK as a possible therapeutic target for Alzheimer’s disease^42^. At the same time, p38 MAPK inhibition also plays protective effect in the cardiovascular disease^43^,^44^. We first found that increased hippocampal p-p38 MAPK expression in HT mice, which was significantly reduced in p38^KI/+^ &HT mice, furthermore, impaired hippocampal synaptic plasticity and memory were improved in p38^KI/+^ &HT mice, and LTP deficit in HT mice was rescued by treatment with p38MAPK inhibitor.

We also found that blood pressure was partially reduced in p38^KI/+^ &HT mice compared with untreated HT mice. This result was similar to previous study which used p38 MAPK inhibitor in diet^14^. While the lower blood pressure may reflect a milder cerebral damage, we think that p38 MAPK knockdown was a major cause for the improvement of synaptic plasticity and cognitive function in p38^KI/+^ &HT mice. There are several points to support our idea. Firstly, hypertension persisted at a level of >140 mmHg. Secondly, the impact of antihypertensive treatment on cognition is less clear. Last but not least, impaired hippocampal CA1 LTP in hippocampal slices from hypertensive mice can be rescued by p38 MAPK inhibitor treatment *in vitro*.

In conclusion, we reported that hippocampal p38 MAPK activation, synaptic plasticity impairment and memory disorder in angiotensin II-dependent hypertensive mice. *In vivo*, we found that synaptic plasticity and memory are improved in angiotensin II-dependent p38^KI/+^ hypertensive mice. *In vitro*, impaired LTP was improved in hippocampal slices from hypertensive mice by p38MAPK inhibitor treatment. Those data indicate that p38MAPK inhibition effectively improves synaptic plasticity and memory in mouse model of hypertension. This study provides novel insights into the mechanisms of hypertension-related cognitive dysfunction and suggests new treatment strategies for counteracting the cognitive impairment of hypertension.

## Methods

Study protocol was approved by the Ethics Committee of Kunming Medical University. All procedures were performed in accordance with our Institutional Guidelines for Animal Research and the investigation conformed to the Guide for the Care and Use of Laboratory Animals published by the US National Institutes of Health (NIH Publication No. 85–23, revised 2011).

### Experimental design

*In vivo* experiment, four experimental groups were analyzed: (1) 0.9% saline solution control C57BL/6 mice (wild-type [WT] group); (2) angiotensin II-treated C57BL/6 mice (hypertensive [HT] group); (3) 0.9% saline solution p38^KI/+^ mice (p38^KI/+^ group); and (4) angiotensin II-treated p38^KI/+^ mice (p38^KI/+^ &HT group). *In vitro* experiment, three experimental C57BL/6 groups were analyzed: (1) control + SKF86002 group; (2) hypertension + vehicle group; (3) hypertension + SKF86002 group.

### Animals

Eight week-old C57BL/6 wild-type male mice and p38^KI/+^ male mice (22–26 g) were used for this study. p38^KI/+^ mice in C57BL/6 background have been described previously (in which activating phosphorylation sites Thr180 and Tyr182 are mutated, leading to an inactivation of phosphorylation of these sites.)^45^, which were obtained from Institute of Molecular and Cell Biology (Biopolis Drive, Proteos, Singapore). Animals were kept under a 12 h/12 h light/dark cycle with the light on at 07:00 AM. They were fed with standard chow and water ad libitum. Ambient temperature and relative humidity were maintained at 22 ± 2 °C and 55 ± 5%, respectively.

### Angiotensin II-induced hypertension

Mice were anaesthetized with intraperitoneal administration of pentobarbital sodium (1.0%, 80 mg/kg). The skin was cleaned with 70% ethanol between the ears and a small skin incision was made to insert an osmotic pump under the skin in the lower back region. After surgery, stitches were used to hold the two edges of tissue together, so the wound could heal. Each osmotic pump (Alzet model 1004) delivered 0.11 μl/h for a 28 day period and was filled with either 1900 ng/kg/min of angiotensin II or a 0.9% saline solution.

### Blood pressure measurement

Blood pressure was taken by non-invasive tail-cuff plethysmography (IITC Life Science, USA). The measurements were taken five days before surgery to accustom the mice to the procedure. Afterwards, blood pressure was measured once a week until the end of the experiment. The animals were displaced in the blood pressure measurement room at least one hour before measurements. All measurements were taken between 8 AM and noon. For each animal, four valid measurements for each time point. Data were not considered valid by the software in the following situations: 1) blood flow was insufficient in the tail; 2) the overall shape of the trace did not fit the expected profile; 3) the diastolic and systolic readings occurred too far apart or too close together.

### Western blot analysis

Hippocampal was homogenized in RIPA buffer (150 mM NaCl, 1.0% NP-40, 0.5% sodium deoxycholate, 0.1% SDS, 50 mM Tris-HCL, PH 8.0) containing protease inhibitor and phosphatase inhibitor (Thermo). Lysates were then dissolved in 2 X laemmli sample buffer (Biorad), and boiled at 95 °C for 5 minutes. For western blot, lysates (50 ug protein) were added in SDS-PAGE and electroblotted onto PVDF membrane. The membranes were blocked with 5% skim milk in TBS-T, and then probed with desired antibodies. Primary antibodies against p-p38 (Cell Signaling), p38(Anbo) and β-actin (Sigma) were used. Immobilon Western Chemiluminescent HRP Substrate (Merck) was used to reveal the antibody-antigen complexes.

### Golgi staining

Mice were processed and stained according to the protocol provided by the manufacture (FD Neuro Technologies). In brief, after brain removal, hemispheres were immersed in Golgi-Cox staining solution for 14d at room temperature in the dark with one change of solution after 24 h. Brains were transferred to C solution at 4 °C for 72 h with one change of solution after 24 h. Coronal sections (150 μm) were mounted onto slides coated with C solution. After drying in the dark, slides were immersed into dH_2_O two times for 2 min and then transferred to a developing solution for 10 min. Slides were then rinsed two times for 2 min in dH_2_O, dehydrated through graded ethanol, immersed into dimethylbenzene two times for 4 min and then coverslipped using resinene. Golgi-stained sections were imaged with an confocal microscopy (Leica, Germany) and used the LAS AF Lite of the imaging software (Leica). Measurements of dendritic morphology were performed on neurons randomly chosen in the CA1 region of the hippocampus for analysis. Two-order branches of apical dendrites from pyramidal neurons in hippocampal CA1 were acquired. At least 5 branches from 5 different neurons were analyzed per animal and calculated the density of spines per 30 μm dendrite.

### Hippocampal slice preparation

Mice were anaesthetized and decapitated. Acute hippocampal slices were cut 350 μm thick using a vibratome (WPI). The slices were gently transferred to a submersion holding chamber containing ACSF (mM: NaCl 126, KCl 2.5, NaH_2_PO4 1, CaCl_2_ 2.5, MgSO_4_ 1.5, NaHCO_3_ 26, and glucose 10) bubbled with 95% O_2_ and 5% CO_2_. Slices were recovered at room temperature for at least 1 h before being transferred to a submersion recording chamber (PSMI; Harvard Apparatus, Holliston, MA, USA), in which the slice were continually perfused with oxygenated ACSF at a rate 1–2 ml/min at room temperature.

### Electrophysiological recording

Field excitatory postsynaptic potentials (fEPSPs) were recorded in the stratum radiatum of the hippocampal CA1 region by means of a glass microelectrode filled with 3 M NaCl (resistance 1–4 MΩ). The Schaffer collateral pathway was stimulated with concentric bipolar electrodes (Frederick Haer Co, Bowdoinham, ME, USA). After an optimal fEPSP wave was found, an input–output (I/O) curve was established with stimulating pulses (0.2 ms duration) at different intensities. Baseline fEPSP was recorded at 0.033 Hz with a stimulating strength adjusted to yield about 40% of the maximal response. After baseline responses had stably lasted for at least 30 min, LTP was induced by delivering theta burst stimuli (TBS; four trains of 10 bursts of four stimuli with 20 s, 200 ms, and 10 ms intervals between trains, bursts, and stimuli, respectively). The electrophysiological data was acquired with a multiclamp 700 A amplifier (Axon instruments, Molecular Devices, USA), filtered at 0.1–5 KHz, digitized at 10 KHz, and analyzed with Clampfit version 10.0 (Axon Instruments, USA). The analyzed data was further processed with Origin 5.1 (Microcal Software Northampton, MA).

### p38 MAPK inhibitor application *in vitro*

p38 MAPK inhibitor SKF86002 was purchased from Sigma. It was dissolved in 100% dimethylsulfoxide (DMSO) stock solution and stored at −20 °C until the day of the experiment. The final concentration of DMSO was kept at 0.02%. SKF86002 (2 μM was used at final concentration) was preperfused over the slices for 10 min before LTP induction.

### Morris water maze

The Morris water maze (MWM) was used for testing hippocampus-dependent spatial memory. It was conducted following the protocols reported previously with minor modification^26^. In brief, animal activity was measured by a video-based tracking system (ANY-maze, Stoelting). The pool was filled with opaque water (by adding washable white paint) and surrounded by extramaze cues. The escape platform (10 cm in diameter) was placed in the center of a designated quadrant with its top positioned 1 cm below the water surface. The light was dimmed and the water temperature kept at 23 ± 2 °C degree. The training phase lasted 4 days during which the mice were allowed four 90 second trials per day to find the escape platform. Each trial was 20 min apart. The probe and cue tests were administered on the 5th day. During the probe test, the hidden platform was removed from the pool, and mice were allowed to swim for 60 sec. The time spent in each quadrant, as well as the number of platform crossings were recorded. The platform was then placed, some water was removed so that the escape platform became visible. One trial was given to evaluate sight and motricity. Sight was measured by the latency finding the escape platform and motricity was assessed by the speed of the mice during the trial.

### Statistical analysis

All data are expressed as mean ± SEM. The data on BPs, electrophysiology and distance and latency of MWM were analyzed by two-way repeated-measures ANOVA. One-way ANOVA was used to analyse other datas. Bonferroni multiple-comparison test was performed for comparisons between groups. Values of P < 0.05 were considered statistically significant. Statistical analysis was performed with SPSS 17.0 (SPSS Inc).

## Additional Information

**How to cite this article**: Dai, H.-L. *et al*. p38 MAPK Inhibition Improves Synaptic Plasticity and Memory in Angiotensin II-dependent Hypertensive Mice. *Sci. Rep.* **6**, 27600; doi: 10.1038/srep27600 (2016).

## References

[b1] Shimamoto K. . The Japanese Society of Hypertension Guidelines for the Management of Hypertension (JSH 2014). Hypertens Res 37, 253–390 (2014). 24705419 10.1038/hr.2014.20

[b2] Yaffe K. . Early adult to midlife cardiovascular risk factors and cognitive function. Circulation 129, 1560–1567 (2014). 24687777 10.1161/CIRCULATIONAHA.113.004798 PMC4700881

[b3] Böhm M. . Systolic blood pressure variation and mean heart rate is associated with cognitive dysfunction in patients with high cardiovascular risk. Hypertension 65, 651–661 (2015). 25583157 10.1161/HYPERTENSIONAHA.114.04568

[b4] Fuster V. Top 10 cardiovascular therapies and interventions for the next decade. Nat Rev Cardiol 11, 671–683 (2014). 25267423 10.1038/nrcardio.2014.137

[b5] Mancia G. . 2013 ESH/ESC Guidelines for the management of arterial hypertension: the Task Force for the management of arterial hypertension of the European Society of Hypertension (ESH) and of the European Society of Cardiology (ESC). J Hypertens 31, 1281–1357 (2013). 23817082 10.1097/01.hjh.0000431740.32696.cc

[b6] Faraco G. & Iadecola C. Hypertension: a harbinger of stroke and dementia. Hypertension 62, 810–817 (2013). 23980072 10.1161/HYPERTENSIONAHA.113.01063 PMC3847558

[b7] Miller E. C. . Differential modulation of drug-induced structural and functional plasticity of dendritic spines. Mol Pharmacol 82, 333–343 (2012). 22596350 10.1124/mol.112.078162 PMC3400837

[b8] Stuchlik A. Dynamic learning and memory, synaptic plasticity and neurogenesis: an update. Front Behav Neurosci 8, 106 (2014). 24744707 10.3389/fnbeh.2014.00106 PMC3978286

[b9] Trinchese F. . Inhibition of calpains improves memory and synaptic transmission in a mouse model of Alzheimer disease. J Clin Invest 118, 2796–2807(2008). 18596919 10.1172/JCI34254 PMC2441853

[b10] Umemoto S. . Different effects of amlodipine and enalapril on the mitogen-activated protein kinese/extraeellular signal-regulated kinase kinase-extracellular signal-regulated kinase pathway for induction of vascular smooth muscle cell differentiation *in vivo*. Hypertens Res 29, 179–186 (2006). 16755153 10.1291/hypres.29.179

[b11] Sun A., Liu M., Nguyen X. V. & Bing G. P38 MAP kinase is activated at early stages in Alzheimer’s disease brain. Exp Neurol 183, 394–405 (2003). 14552880 10.1016/s0014-4886(03)00180-8

[b12] Munoz L. . A novel p38 alpha MAPK inhibitor suppresses brain proinflammatory cytokine up-regulation and attenuates synaptic dysfunction and behavioral deficits in an Alzheimer’s disease mouse model. J Neuroinflammation 4, 21 (2007). 17784957 10.1186/1742-2094-4-21 PMC2014744

[b13] Corrêa S. A. & Eales K. L. The Role of p38 MAPK and Its Substrates in Neuronal Plasticity and Neurodegenerative Disease. J Signal Transduct 2012, 649079 (2012). 22792454 10.1155/2012/649079 PMC3389708

[b14] Park J. K. . p38 mitogen-activated protein kinase inhibition ameliorates angiotensin II-induced target organ damage. Hypertension 49, 481–489(2007). 17224470 10.1161/01.HYP.0000256831.33459.ea

[b15] Wu J. . Inflammation and mechanical stretch promote aortic stiffening in hypertension through activation of p38 mitogen-activated protein kinase. Circ Res 114, 616–625 (2014). 24347665 10.1161/CIRCRESAHA.114.302157 PMC4186716

[b16] Swatton J. E. . Increased MAP kinase activity in Alzheimer’s and Down syndrome but not in schizophrenia human brain. Eur J Neurosci 19, 2711–2719 (2014). 15147305 10.1111/j.0953-816X.2004.03365.x

[b17] Savage M. J., Lin Y. G., Ciallella J. R., Flood D. G. & Scott R. W. Activation of c-Jun N-terminal kinase and p38 in an Alzheimer’s disease model is associated with amyloid deposition. J Neurosci 22, 3376–3385 (2002). 11978814 10.1523/JNEUROSCI.22-09-03376.2002 PMC6758401

[b18] Jin Y. . Sodium ferulate prevents amyloid-beta-induced neurotoxicity through suppression of p38 MAPK and upregulation of ERK-1/2 and Akt/protein kinase B in rat hippocampus. Acta Pharmacol Sin 26, 943–951 (2005). 16038626 10.1111/j.1745-7254.2005.00158.x

[b19] Wang Q., Walsh D. M., Rowan M. J., Selkoe D. J. & Anwyl R. Block of long-term potentiation by naturally secreted and synthetic amyloid beta-peptide in hippocampal slices is mediated via activation of the kinases c-Jun N-terminal kinase, cyclin-dependent kinase 5, and p38 mitogen-activated protein kinase as well as metabotropic glutamate receptor type 5. J Neurosci 24, 3370–3378 (2004). 15056716 10.1523/JNEUROSCI.1633-03.2004 PMC6730034

[b20] Matsumoto A. . Day-to-day variability in home blood pressure is associated with cognitive decline: the Ohasama study. Hypertension 63, 1333–1338 (2014). 24688128 10.1161/HYPERTENSIONAHA.113.01819

[b21] Gottesman R. F. . Midlife hypertension and 20-year cognitive change: the atherosclerosis risk in communities neurocognitive study. JAMA Neurol 71, 1218–1227 (2014). 25090106 10.1001/jamaneurol.2014.1646 PMC4226067

[b22] Köhler S. . Temporal evolution of cognitive changes in incident hypertension: prospective cohort study across the adult age span. Hypertension 63, 245–251 (2014). 24296281 10.1161/HYPERTENSIONAHA.113.02096

[b23] Hartman R. E., Kamper J. E., Goyal R., Stewart J. M. & Longo L. D. Motor and cognitive deficits in mice bred to have low or high blood pressure. Physiol Behav 105, 1092–1097 (2012). 22154805 10.1016/j.physbeh.2011.11.022

[b24] Gentile M. T. . β-Amyloid deposition in brain is enhanced in mouse models of arterial hypertension. Neurobiol Aging 30, 222–228 (2009). 17673335 10.1016/j.neurobiolaging.2007.06.005

[b25] Carnevale D. . Hypertension induces brain β-amyloid accumulation, cognitive impairment, and memory deterioration through activation of receptor for advanced glycation end products in brain vasculature. Hypertension 60, 188–197 (2012). 22615109 10.1161/HYPERTENSIONAHA.112.195511 PMC3530195

[b26] Duchemin S., Belanger E., Wu R., Ferland G. & Girouard H. Chronic perfusion of angiotensin II causes cognitive dysfunctions and anxiety in mice. Physiol Behav 109, 63–68 (2013). 23103834 10.1016/j.physbeh.2012.10.005

[b27] Wang M. H., Chang W. J., Soung H. S. & Chang K. C. (−)-Epigallocatechin-3-gallate decreases the impairment in learning and memory in spontaneous hypertension rats. Behav Pharmacol 23, 771–780 (2012). 23044831 10.1097/FBP.0b013e32835a3bc8

[b28] Mizuno K., Wakai M., Takeda A. & Sobue G. Medial temporal atrophy and memory impairment in early stage of Alzheimer’s disease: an MRI volumetric and memory assessment study. J Neurol Sci 173, 18–24 (2000). 10675575 10.1016/s0022-510x(99)00289-0

[b29] Haag M. D., Hofman A., Koudstaal P. J., Breteler M. M. & Stricker B. H. Duration of antihypertensive drug use and risk of dementia: A prospective cohort study. Neurology. 72, 1727–1734 (2009). 19228584 10.1212/01.wnl.0000345062.86148.3f

[b30] Hajjar I., Brown L., Mack W. J. & Chui H. Impact of Angiotensin receptor blockers on Alzheimer disease neuropathology in a large brain autopsy series. Arch Neurol 69, 1632–1638 (2012). 22964777 10.1001/archneurol.2012.1010 PMC3608189

[b31] Li N.-C. . Use of angiotensin receptor blockers and risk of dementia in a predominantly male population: prospective cohort analysis. BMJ 340, b5465 (2010). 20068258 10.1136/bmj.b5465 PMC2806632

[b32] Anderson C. . Renin-angiotensin system blockade and cognitive function in patients at high risk of cardiovascular disease: analysis of data from the ONTARGET and TRANSCEND studies. Lancet Neurol 10, 43–53 (2011). 20980201 10.1016/S1474-4422(10)70250-7

[b33] Williamson J. D. . Cognitive function and brain structure in persons with type 2 diabetes mellitus after intensive lowering of blood pressure and lipid levels: a randomized clinical trial. JAMA Intern Med 174, 324–333 (2014). 24493100 10.1001/jamainternmed.2013.13656 PMC4423790

[b34] Peters R., Booth A. & Peters J. A systematic review of calcium channel blocker use and cognitive decline/dementia in the elderly. J Hypertens 32, 1945–1957 (2014). 25068540 10.1097/HJH.0000000000000273

[b35] Beishon L. C. . The evidence for treating hypertension in older people with dementia: a systematic review. J Hum Hypertens 28, 283–287 (2014). 24196416 10.1038/jhh.2013.107

[b36] Richmond R., Law J. & Kay-Lambkin F. Higher blood pressure associated with higher cognition and functionality among centenarians in Australia. Am J Hypertens 24, 299–303 (2011). 21164496 10.1038/ajh.2010.236

[b37] Szewieczek J. . Better cognitive and physical performance is associated with higher blood pressure in centenarians. J Nutr Health Aging 15, 618–622 (2011). 21968855 10.1007/s12603-011-0334-8 PMC12878851

[b38] Cifuentes D. . Hypertension accelerates the progression of Alzheimer-like pathology in a mouse model of the disease. Hypertension 65, 218–224 (2015). 25331846 10.1161/HYPERTENSIONAHA.114.04139

[b39] Kruyer A., Soplop N., Strickland S. & Norris E. H. Chronic Hypertension Leads to Neurodegeneration in the TgSwDI Mouse Model of Alzheimer’s Disease. Hypertension 66, 175–182 (2015). 25941345 10.1161/HYPERTENSIONAHA.115.05524 PMC4465852

[b40] Salloway S. . Two phase 3 trials of bapineuzumab in mild-to-moderate Alzheimer’s disease. N Engl J Med 370, 322–333 (2014). 24450891 10.1056/NEJMoa1304839 PMC4159618

[b41] Doody R. S. . Phase 3 trials of solanezumab for mild-to-moderate Alzheimer’s disease. N Engl J Med 370, 311–321 (2014). 24450890 10.1056/NEJMoa1312889

[b42] Dalrymple S. A. p38 mitogen activated protein kinase as a therapeutic target for Alzheimer’s disease. J Mol Neurosci 19, 295–299(2012). 12540055 10.1385/JMN:19:3:295

[b43] Cheriyan J. . Inhibition of p38 mitogen-activated protein kinase improves nitric oxide-mediated vasodilatation and reduces inflammation in hypercholesterolemia. Circulation 123, 515–523(2011). 21262998 10.1161/CIRCULATIONAHA.110.971986

[b44] Newby L. K. . Losmapimod, a novel p38 mitogen-activated protein kinase inhibitor, in non-ST-segment elevation myocardial infarction: a randomised phase 2 trial. Lancet 384, 1187–1195 (2014). 24930728 10.1016/S0140-6736(14)60417-7

[b45] Wong E. S. . p38MAPK controls expression of multiple cell cycle inhibitors and islet proliferation with advancing age. Dev Cell 17, 142–149 (2009). 19619499 10.1016/j.devcel.2009.05.009

